# Supplementary material for: P-TRAP: a Panicle Trait Phenotyping tool
Source: BMC Plant Biol. 2013 Aug 29;13:122. doi: 10.1186/1471-2229-13-122 (PMC3848748; doi:10.1186/1471-2229-13-122)
Supplement: Additional file 7 — P-TRAP source code. The Netbeans project that contains the source code of the application. [file 1471-2229-13-122-S7.zip › javadoc.zip!/javadoc/org-bioalg-graphprocessor/org/bioalg/graphprocessor/GraphModel.html]

GraphModel (GraphProcessor)


JavaScript is disabled on your browser.


- Package
- Class
- Tree
- Deprecated
- Index
- Help

*org.bioalg.graphprocessor 1.0*

- Prev Class
- Next Class

- Frames
- No Frames

- All Classes

- Summary:
- Nested |
- Field |
- Constr |
- Method

- Detail:
- Field |
- Constr |
- Method


org.bioalg.graphprocessor

## Class GraphModel

- java.lang.Object
- - org.bioalg.graphprocessor.GraphModel

- ---

    

  ```
  public class GraphModel
  extends java.lang.Object
  ```

  A graph model that can be used to warp xml files, this is more portable than the `Graph` class for both visualizing the results and user manual editing.

  Author:
  :   faroq

- - ### Nested Class Summary

    Nested Classes

    | Modifier and Type | Class and Description |
    | --- | --- |
    | `static class` | `GraphModel.GraphErrors` |
  - ### Constructor Summary

    Constructors

    Constructor and Description

    `GraphModel()`

    `GraphModel(org.w3c.dom.Element graph)`

    Constructor from an XML element.

    `GraphModel(java.io.InputStream xmlstring)`

    Constructor from an `InputStream` object.
  - ### Method Summary

    Methods

    | Modifier and Type | Method and Description |
    | --- | --- |
    | `void` | `addEdge(Vertex vertex1, Vertex vertex2)` |
    | `void` | `addvetex(Vertex vertex)` |
    | `void` | `clearall()` |
    | `void` | `fixPrimaryJunctions()` |
    | `java.util.ArrayList<BranchModel>` | `getAllBranches()` |
    | `java.util.ArrayList<EdgeModel>` | `getEdges()` |
    | `java.util.ArrayList<Vertex>` | `getfixedPoints()` |
    | `java.util.ArrayList<Vertex>` | `getNeighbors(Vertex vertex)` |
    | `java.util.ArrayList<Vertex>` | `getNeighbors(Vertex vertex, Vertex.VERTEX_TYPE type)` |
    | `java.util.ArrayList<Vertex>` | `getNeighbors(Vertex vertex, Vertex.VERTEX_TYPE type1, Vertex.VERTEX_TYPE type2)` |
    | `double` | `getRachisLength(java.util.ArrayList<BranchModel> branches)` |
    | `Vertex` | `getvertexByid(java.lang.String id)` |
    | `java.util.ArrayList<Vertex>` | `getVertices()` |
    | `boolean` | `hasChildlessNode()` |
    | `boolean` | `hasFlyingNodes()` |
    | `boolean` | `hasTwoFixedNodes()` |
    | `boolean` | `isdummy(java.util.ArrayList<Vertex> vs)` |
    | `boolean` | `isTransition(Vertex vertex)` |
    | `void` | `makeDirected()` convert it to a directed graph |
    | `void` | `refineModel(int distance)` Remove crowded nodes at the junctions |
    | `void` | `removeEdge(EdgeModel em)` |
    | `void` | `removeVertex(Vertex vertex)` |
    | `void` | `setEdges(java.util.ArrayList<EdgeModel> edges)` |
    | `void` | `setVertices(java.util.ArrayList<Vertex> vertices)` |
    | `void` | `showError(GraphModel.GraphErrors errors)` |
    | `java.lang.String` | `toString()` |
    | `GraphModel.GraphErrors` | `verfiyModel()` |

    - ### Methods inherited from class java.lang.Object

      `clone, equals, finalize, getClass, hashCode, notify, notifyAll, wait, wait, wait`

- - ### Constructor Detail


    - #### GraphModel

      ```
      public GraphModel()
      ```


    - #### GraphModel

      ```
      public GraphModel(java.io.InputStream xmlstring)
      ```

      Constructor from an `InputStream` object.

      Parameters:
      :   `xmlstring` -


    - #### GraphModel

      ```
      public GraphModel(org.w3c.dom.Element graph)
      ```

      Constructor from an XML element.

      Parameters:
      :   `graph` -
  - ### Method Detail


    - #### clearall

      ```
      public void clearall()
      ```


    - #### addEdge

      ```
      public void addEdge(Vertex vertex1,
                 Vertex vertex2)
      ```


    - #### removeEdge

      ```
      public void removeEdge(EdgeModel em)
      ```


    - #### addvetex

      ```
      public void addvetex(Vertex vertex)
      ```


    - #### removeVertex

      ```
      public void removeVertex(Vertex vertex)
      ```


    - #### getvertexByid

      ```
      public Vertex getvertexByid(java.lang.String id)
      ```


    - #### toString

      ```
      public java.lang.String toString()
      ```

      **Overrides:**
      :   `toString` in class `java.lang.Object`


    - #### getEdges

      ```
      public java.util.ArrayList<EdgeModel> getEdges()
      ```


    - #### setEdges

      ```
      public void setEdges(java.util.ArrayList<EdgeModel> edges)
      ```


    - #### getVertices

      ```
      public java.util.ArrayList<Vertex> getVertices()
      ```


    - #### setVertices

      ```
      public void setVertices(java.util.ArrayList<Vertex> vertices)
      ```


    - #### isdummy

      ```
      public boolean isdummy(java.util.ArrayList<Vertex> vs)
      ```


    - #### getNeighbors

      ```
      public java.util.ArrayList<Vertex> getNeighbors(Vertex vertex)
      ```


    - #### getNeighbors

      ```
      public java.util.ArrayList<Vertex> getNeighbors(Vertex vertex,
                                             Vertex.VERTEX_TYPE type)
      ```


    - #### getNeighbors

      ```
      public java.util.ArrayList<Vertex> getNeighbors(Vertex vertex,
                                             Vertex.VERTEX_TYPE type1,
                                             Vertex.VERTEX_TYPE type2)
      ```


    - #### getAllBranches

      ```
      public java.util.ArrayList<BranchModel> getAllBranches()
      ```


    - #### getRachisLength

      ```
      public double getRachisLength(java.util.ArrayList<BranchModel> branches)
      ```


    - #### getfixedPoints

      ```
      public java.util.ArrayList<Vertex> getfixedPoints()
      ```


    - #### isTransition

      ```
      public boolean isTransition(Vertex vertex)
      ```


    - #### hasFlyingNodes

      ```
      public boolean hasFlyingNodes()
      ```


    - #### hasTwoFixedNodes

      ```
      public boolean hasTwoFixedNodes()
      ```


    - #### hasChildlessNode

      ```
      public boolean hasChildlessNode()
      ```


    - #### verfiyModel

      ```
      public GraphModel.GraphErrors verfiyModel()
      ```


    - #### showError

      ```
      public void showError(GraphModel.GraphErrors errors)
      ```


    - #### refineModel

      ```
      public void refineModel(int distance)
      ```

      Remove crowded nodes at the junctions


    - #### fixPrimaryJunctions

      ```
      public void fixPrimaryJunctions()
      ```


    - #### makeDirected

      ```
      public void makeDirected()
      ```

      convert it to a directed graph


- Package
- Class
- Tree
- Deprecated
- Index
- Help

*org.bioalg.graphprocessor 1.0*

- Prev Class
- Next Class

- Frames
- No Frames

- All Classes

- Summary:
- Nested |
- Field |
- Constr |
- Method

- Detail:
- Field |
- Constr |
- Method
